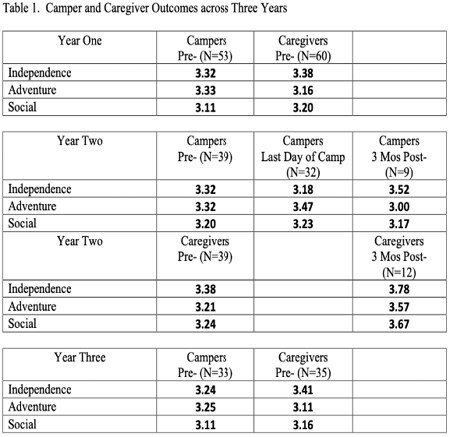# 732 An Evidenced Based Approach to Capturing Growth and Change Through the Burn Camp Experience

**DOI:** 10.1093/jbcr/irae036.275

**Published:** 2024-04-17

**Authors:** Brad Jackson, Kerry Mikolaj, Trudy J Boulter, Nichole Schiffer, Genevieve Kierulf

**Affiliations:** Children's Hospital Colorado, Denver, CO; Children's Hospital Colorado, Aurora, CO; Children's Hospital Colorado, Denver, CO; Children's Hospital Colorado, Aurora, CO; Children's Hospital Colorado, Denver, CO; Children's Hospital Colorado, Aurora, CO; Children's Hospital Colorado, Denver, CO; Children's Hospital Colorado, Aurora, CO; Children's Hospital Colorado, Denver, CO; Children's Hospital Colorado, Aurora, CO

## Abstract

**Introduction:**

The American Camping Association (ACA) explored “in what ways do children change because of camp experiences” (Thurber et al, 2007). Our burn camp approached this same question about the medical specialty camp experience assessing readiness and change in three key areas of growth: Independence / Leadership, Adventure / Exploration, and Social Skills.

**Methods:**

The ACA supported the development of the Camper Growth Index (Henderson et al, 2006). For our medical specialty camp, a subset of items was selected for each area based on the strength of factor scores in the original study, relevance to our camp setting, and the growth being documented in counselor reports.

Campers ages 8 – 18 and caregivers rated items addressing these areas on a 4 point likert scale from disagree to agree. Sample questions are “I am good at doing things on my own”, “I like to talk to kids I don’t know yet”, and “My child likes to try new activities”.

Campers and caregivers received the questionnaires across a 3 year period.

**Results:**

Pre-Camp ratings from campers and caregivers are consistent across the 3 years (Table 1). When campers were queried on the last day of camp, their adventure / exploration scores trended upwards, however that trend did not continue at 3 months, but independence / leadership trended upwards instead. Caregivers rated all three categories higher at the 3 month post camp assessment. Variation in the responses between individual items suggests discriminant validity and that certain items might be more sensitive to capturing the benefits of the burn camp experience.

**Conclusions:**

Burn camps are a vital part of the burn rehab and aftercare experience for young burn survivors and their families. Documenting change through burn camp remains an important task. Change in these growth areas supports our targeted and intentional programming. The Camper Growth Index utilized by ACA allows us to assess the camper and caregiver experience of change, while linking the burn camp experience to the larger youth camp community of research.

**Applicability of Research to Practice:**

We are evaluating the sensitivity of this abbreviated measure within a burn camp setting. Along with other measures of growth and change, we are hoping to document the magic of burn camp.